# Antioxidant Responses in Copepods Are Driven Primarily by Food Intake, Not by Toxin-Producing Cyanobacteria in the Diet

**DOI:** 10.3389/fphys.2021.805646

**Published:** 2022-01-04

**Authors:** Elena Gorokhova, Rehab El-Shehawy

**Affiliations:** Department of Environmental Science, Stockholm University, Stockholm, Sweden

**Keywords:** AChE, antioxidant enzymes, Baltic zooplankton, CAT, feeding and growth indices, molecular diet analysis, GST, SOD

## Abstract

The association between oxidative processes and physiological responses has received much attention in ecotoxicity assessment. In the Baltic Sea, bloom-forming cyanobacterium *Nodularia spumigena* is a significant producer of various bioactive compounds, and both positive and adverse effects on grazers feeding in cyanobacteria blooms are reported. To elucidate the effect mechanisms and species sensitivity to the cyanobacteria-dominating diet, we exposed two Baltic copepods, *Acartia bifilosa* and *Eurytemora affinis*, to a diet consisting of toxin-producing cyanobacteria *N. spumigena* and a high-quality food *Rhodomonas salina* at 0–300 μg C L^−1^; the control food was *R. salina* provided as a monodiet at the same food levels. The subcellular responses to food type and availability were assayed using a suite of biomarkers – antioxidant enzymes [superoxide dismutases (SOD), catalase (CAT), and glutathione S-transferases (GST)] and acetylcholinesterase (AChE). In parallel, we measured feeding activity using gut content (GC) assayed by real-time PCR analysis that quantified amounts of the prey DNA in copepod stomachs. As growth and reproduction endpoints, individual RNA content (a proxy for protein synthesis capacity), egg production rate (EPR), and egg viability (EV%) were used. In both toxic and nontoxic foods, copepod GC, RNA content, and EPR increased with food availability. Antioxidant enzyme activities increased with food availability regardless of the diet type. Moreover, CAT (both copepods), SOD, and GST (*A. bifilosa*) were upregulated in the copepods receiving cyanobacteria; the response was detectable when adjusted for the feeding and/or growth responses. By contrast, the diet effects were not significant when food concentration was used as a co-variable. A bimodal response in AChE was observed in *A. bifilosa* feeding on cyanobacteria, with up to 52% increase at the lower levels (5–25 μg C L^−1^) and 32% inhibition at the highest food concentrations. These findings contribute to the refinement of biomarker use for assessing environmental stress and mechanistic understanding of cyanobacteria effects in grazers. They also suggest that antioxidant and AChE responses to feeding activity and diet should be accounted for when using biomarker profiles in field-collected animals in the Baltic Sea and, perhaps other systems, where toxic cyanobacteria are common.

## Introduction

The health conditions in biota are influenced by numerous environmental factors, with oxidative stress, i.e., the imbalance between pro-oxidant and antioxidant homeostatic cellular conditions, mediating many disorders. Pro-oxidant conditions occur due to either increased generation of reactive oxygen species (ROS) or their poor quenching/scavenging into the body, potentially inducing damage to macromolecules, including proteins lipids, and DNA (Sies et al., 2017). While naturally occurring at low levels, ROS can also be induced by environmental conditions, both natural (e.g., hypoxia, UV radiation, and bioactive compounds) and anthropogenic (e.g., chemical contaminants). Antioxidant defense systems consisting of low molecular-weight antioxidants and antioxidant enzymes have evolved to reduce the pro-oxidation effects. The essential antioxidant enzymes (Pisoschi and Pop, 2015) are (i) ROS eliminating enzymes [e.g., superoxide dismutases (SOD), catalase (CAT), and peroxidases], (ii) enzymes that eliminate internal lipid peroxidation products [e.g., glutathione peroxidases], and (iii) those eliminating toxic secondary radical oxidation products [e.g., glutathione S-transferases (GST)]. A balance between ROS production and antioxidants to quench and/or scavenge them can be tipped by any additional burden of free radicals either from the environment or produced within the body, leading to oxidative stress manifested as fitness penalties.

In ecology and ecotoxicology, the antioxidant enzymes are used as biomarkers to identify natural and anthropogenic stressors and understand the physiological consequences of exposure (Monserrat et al., 2007). However, the interpretation of the measured values is seldom straightforward, with numerous difficulties related to the non-specificity of the antioxidant responses and considerable variability due to various biological and environmental factors (Cohen et al., 2009). Furthermore, feeding can also modulate these responses as high metabolic activity is associated with elevated ROS production (Sohal and Weindruch, 1996; Furuhagen et al., 2014), and suboptimal food quality may contribute to pro-oxidative processes (Zenteno et al., 2008). Therefore, assessing antioxidant biomarkers in concert with metabolic variables, such as feeding, respiration, and growth, would facilitate biomarker interpretation.

Naturally occurring bioactive compounds, such as cyanobacterial secondary metabolites, can induce oxidative stress and activate antioxidant defenses in eukaryotes (Ding and Ong, 2003; Wiegand and Pflugmacher, 2005; Amado and Monserrat, 2010). Moreover, filamentous cyanobacteria are commonly considered inadequate food because of (i) cyanotoxins, (ii) poor manageability, and (iii) low nutritional value due to low content of polyunsaturated fatty acids and sterols (Gulati and DeMott, 1997). Therefore, in zooplankton feeding on cyanobacteria, the antioxidant responses may be related to oxidative stress caused by various bioactive compounds, decreased metabolic activity due to the low food intake/assimilation, and poor nutrition value (Bednarska et al., 2014). Moreover, the variations in the overall food availability, food intake, and growth would also contribute to the redox status in these grazers (Furuhagen et al., 2014).

The enzymes SOD, CAT, and GST are commonly used for oxidative status assessment in fish (Jos et al., 2005; Persson et al., 2009) and invertebrates, e.g., mussels (Davies et al., 2005; Kankaanpää et al., 2007), crabs (Pinho et al., 2003), and copepods (Kozlowsky-Suzuki et al., 2009), exposed to cyanobacterial hepatotoxins (microcystin and nodularin). Moreover, in fish and invertebrates, cyanobacterial neurotoxins have been implicated in inhibiting acetylcholinesterase (AChE; Monserrat et al., 2007), the hydrolyzing enzyme of the neurotransmitter acetylcholine in cholinergic synapses. As various organophosphates and carbamates inhibit AChE, it is a well-established biomarker of pesticide exposure (Lionetto et al., 2013). Therefore, any non-specific alterations in its activity related to food intake or growth are of particular concern. Thus, the very same set of enzymes is used to assess the effects of pollution in systems, where co-exposure to cyanobacterial blooms is common, such as the Baltic Sea (Kopecka et al., 2006; Lehtonen et al., 2006; Napierska et al., 2009).

Cyanobacterial blooms are a regular feature of the Baltic Sea. *Nodularia spumigena*, *Aphanizomenon* spp., and *Dolichospermum* spp. are the main contributors to these blooms. *Nodularia spumigena* is usually the primary concern because of the cyclic pentapeptide nodularin, a protein phosphatase inhibitor similar to microcystins (Sivonen and Börner, 2008), and a broad spectrum of other metabolically active compounds (Lage et al., 2021). Virtually all pelagic and benthic species are exposed to these toxic outbreaks, which often coincides with low growth and fecundity (Koski et al., 1999; Engström-Öst et al., 2015). Most laboratory studies of mesozooplankton grazing on natural plankton assemblages show avoidance of cyanobacteria and preferential feeding on non-toxic strains (Lampert, 1987; Sellner et al., 1996). However, the evidence is accumulating that cyanobacteria contribute substantially to the diets of some copepods and mysids *in situ* (Kozlowsky-Suzuki et al., 2003; Peters et al., 2006; Gorokhova, 2009; Motwani et al., 2017; Gorokhova et al., 2021). Coevolutionary histories, regular exposure, and high ingestion of toxic cyanobacteria support the idea that these animals might cope with ingested toxins. Moreover, toxin biodegradation of the copepod gut microbiome has been recently proposed as a mechanism behind successful feeding and growth during cyanobacterial blooms (Gorokhova et al., 2021). However, this does not mean that chronic exposure to the high abundances of the toxin-producing cyanobacteria induces no adverse sublethal effects.

Here, we tested the effects of exposure to the filamentous cyanobacterium *N. spumigena* in the copepods *Acartia bifilosa* and *Eurytemora affinis*, the dominant zooplankton species co-occurring with the cyanobacterium (Johansson et al., 2004). These copepods cope relatively well with the cyanobacteria-rich diets (Koski et al., 1999; Vehmaa et al., 2013); moreover, nodularin has been reported to induce antioxidative defenses and combat the oxidative damage, thus contributing toward maintenance of the oxidative balance in these copepods (Vehmaa et al., 2013). In line with this, low toxin retention (Guisande et al., 2002; Karjalainen et al., 2003; Kozlowsky-Suzuki et al., 2009) suggests that fairly efficient detoxification mechanisms may exist in these and, probably, other animals co-existing with toxic blooms, making them relevant as test species for ecotoxicological surveys. However, we expected to find stronger diet effects in *A. bifilosa* than *E. affinis* because of the higher sensitivity of the former to the cyanobacteria diet manifested as adverse effects on growth and reproduction reported in the field and laboratory studies (Motwani et al., 2017; Gorokhova et al., 2021).

## Materials and Methods

Feeding experiments with the copepods fed with cyanobacterium-rich diet and adequate non-toxic food control were conducted to assess the effects of *N. spumigena* on suborganismal responses [antioxidant enzymes (SOD, CAT, and GST) and neurotoxicity biomarker AChE], food intake, and growth. The *N. spumigena* concentrations in the experiment corresponded to its ambient abundances in the northern Baltic proper (Hajdu et al., 2007). The food density gradient was created, and copepod feeding and growth at different food concentrations were assayed using a set of proxies to evaluate how the enzyme activities change with increasing food intake and growth status. Copepod stomach content was analyzed by real-time quantitative PCR (qPCR) with prey-specific primers (Engström-Öst et al., 2011) to assess the amount of specific food ingested. As growth proxies, individual RNA content (Höök et al., 2008; Holmborn et al., 2009) and egg production rate (EPR) were used for somatic and reproductive growth. In addition to EPR, egg viability (VE) was determined (Gorokhova, 2010) to indicate diet inadequacy and possible reproductive toxicity.

### Sampling and Sorting of Copepods

Copepods for the experiments were collected in the Himmerfjärden Bay, in the north-western Baltic proper (58°59΄N 17°44΄E) in the first week of June 2008, before the appearance of *Nodularia spumigena* in the water column. Zooplankton samples were taken from the upper 10 m by vertical hauls using a WP-2 net (mesh size 200 μm, ∅ 57 cm) equipped with a cod-end. Surface water temperature during sampling was 14°C, and salinity was 6.2 ‰. The tow contents were placed in large (~20 L) insulated containers, diluted with surface water, and brought to the lab within a few hours. The containers were gently aerated until sorting commenced. With a light-trap, we separated zooplankton from phytoplankton and filtered onto 500 μm sieve to retain mostly older copepodites (CIII-CVI) of *A. bifilosa* and *E. affinis*. The contribution of the developmental stages at the time of sampling was similar between the species, with CIII contributing <10% in both copepods and CVI being the most common stage (~50% in *A. bifilosa* and 60% in *E. affinis*); the sex ratio (male/female; based on CV and CIV animals) was 0.25 in *A. bifilosa* and 0.36 in *E. affinis*. The copepods were then washed into a Petri dish and sorted under a dissecting microscope (Leica, 40×), using a wide-mouth pipette.

### Cyanobacterial and Algal Cultures

Cultures were maintained in extended exponential growth through a semi-continuous harvesting regime (~30% exchange every third day) at 15°C and 6.5 ‰ salinity in artificial seawater (ASW; Instant Ocean™, Aquarium Systems) with constant illumination (~90 μmol photon PAR m^−2^ s^−1^). The cyanobacterium *Nodularia spumigena*, strain AV1, was grown in a modified Z8 nutrient solution (Sivonen et al., 1989). The largest and least edible colonies were removed by filtering on a 63-μm sieve. The abundance of *N. spumigena* in the feeding media was adjusted using cell counts for stock samples (5 ml) preserved with acid Lugol’s solution and counted as 100-μm filaments (~200 units per sample) in Utermöhl chamber (Fluovert microscope, 125×); the counts were converted to carbon mass (C, μg L^−1^) according to Olenina et al. (2006) and carbon content of 0.11 pg C μm^−3^. The alga used as a high-quality food was *Rhodomonas salina*, strain CCAP 978/24, grown on *f*/20 medium. The algal concentrations (cells ml^−1^) and cell size were determined using a laser particle counter, Spectrex PC-2000 (Spectrex Corp., California), and converted to carbon mass (46 pg C cell^−1^) according to (Mullin et al., 1966).

### Experimental Setup

A mixed community containing ~130 older copepodites of both species (CIII-CVI, prosome length 510 ± 20 and 525 ± 22 μm in *Acartia* and *Eurytemora*, respectively; mean ± SD; *N* = 20) per replicate was used. Both species contributed approximately equally in each experimental unit; *Eurytemora* females with egg sacks were not included. The incubations were carried out in 5 L plastic containers with dilutions of *N. spumigena* and *R. salina* cultures to nominal concentrations of 5, 25, 125, and 300 μg C L^−1^ in 0.2-μm filtered seawater (FSW). The copepods were fed with either a control monodiet of *R. salina* or a mixed diet of *N. spumigena* and *R. salina* (60:40 by carbon mass); this mixture approximates cyanobacteria contribution to the Baltic phytoplankton community in the trophogenic layer during a very heavy summer bloom (Hogfors et al., 2014). Five replicates were set for each treatment, i.e., a combination of food type (i.e., *N. spumigena* present/absent) and food concentration. Copepods incubated in FSW served as starved control; these individuals were also used as negatives in the qPCR analysis. All experimental containers were provided with gentle aeration in a temperature-controlled room (15°C) for ~40 h and 16^L^:8^D^ light cycle; pH was monitored daily (8.2 ± 0.2).

To control for food depletion, a 20-ml sample of the media was taken from each container every 6–8 h; these samples were pooled within a treatment assuming a similar change in the prey concentrations. The algal and cyanobacterial cell concentrations were determined as described above and compared to the nominal concentrations in the respective treatments; if prey depletion was >5%, the food levels were reconstituted by adding the respective foods to the experimental units at the amounts required to reach the nominal concentration. During both experiments, the food concentrations decreased by no more than 17% between the checkpoints.

### Sampling

Upon the experiment termination, the contents of the 5-L containers were sequentially filtered through submerged 200-μm (to collect copepods) and 35-μm (to collect *Acartia* eggs) sieves. The copepods were rinsed twice with FSW, inspected for mortalities, and immobilized with 0.6 g L^−1^ of MS222. When present, egg sacks were separated from *Eurytemora* females, pooled, transferred to a depression slide (one slide per replicate), and egg number was counted using Fluovert microscope, 125×. Under a dissecting microscope, 35–40 copepods of each species from each replicate were transferred to a 1.5-ml Eppendorf tube for enzyme activity measurements and snap frozen at −86°C. Of the remaining copepods, two bulk samples were taken: 5 immature females (CIV) were preserved with 100 μl of RNA*later* for RNA measurements (Gorokhova, 2005), and 5 adult females (CV-CVI) were snap-frozen for GC qPCR analysis. All samples were kept on ice while sorting.

### Egg Production and Viability

Egg production estimates (EPR, eggs females^−1^) were based on the number of eggs retained on the sieve (*Acartia*) or counted in the egg sacks (*Eurytemora*), and the number of the adult females (CVI) recovered. To assay egg viability, eggs were stained using TO-PRO-1 iodide (Gorokhova, 2010). In replicates with <30 eggs, all eggs were used for staining, whereas in replicates with >30 eggs, a random batch of 30 ± 4 (mean ± SD) eggs was counted using an epifluorescence microscope with a blue filter (Leica DM IRB, ×100). The viability was calculated as the percentage of viable eggs (VE%).

### Enzyme Assays

Each sample was homogenized (20% wt/vol) in a cold (4°C) phosphate-buffered saline (PBS), pH 7.4, containing 1% Triton X-100, for 2 min using FastPrep homogenizer. An aliquot of the homogenate was used for the determination of the protein content, and the remaining part was centrifuged at 15000 *g* (15 min, 4°C). The supernatant was used for enzyme activity assays; all assays were performed in duplicates.

The AChE activity was measured with the colorimetric assay (Ellman et al., 1961) adapted for a microplate format (Bocquené and Galgani, 1998) using a microplate reader FLUOstar Optima (BMG Labtechnologies) with absorbance configuration. Acetylthiocholine iodide (AcSCh) was used as a substrate and dithiobisnitrobenzoate (DTNB) as a reagent. To inhibit non-specific cholinesterase activity, ethopropazine-HCl (10^−4^ M, Sigma) was added to the incubation mixture. The rate of change of absorbance at 412 nm due to the enzyme activity was recorded over 1.5 min (20°C) and expressed as nmol of AcSCh hydrolyzed min^−1^ mg^−1^ protein.

The SOD activity was measured using the spectrophotometric assay (Flohé and Ötting, 1985) adapted for a microplate format (Marie et al., 2006). The reduction rate of 2 μM cytochrome *c* was measured at 550 nm (20°C), in 180 μl of phosphate buffer (50 mM, pH = 7.8) with 0.5 mmol EDTA, 5 μM hypoxanthine and 10 μl of the supernatant using FLUOstar Optima with absorbance configuration. The reaction was initiated by injecting 10 μl xanthine oxidase (0.2 U ml^−1^). SOD activity in samples was estimated with Cu, Zn-SOD purified from bovine erythrocytes (Sigma) and expressed as U mg protein^−1^ (1 U corresponds to the SOD amount inhibiting by 50% the rate of cytochrome *c* reduction).

The CAT activity was measured using the microplate chemiluminescence assay (Maral et al., 1977; Janssens et al., 2000) and FLUOstar Optima with luminescence configuration. The consumption of H_2_O_2_ by CAT was followed when 20 μl of 1 μM H_2_O_2_ were added to 20 μl of sample diluted in 80 μl of phosphate buffer (100 mM; pH 7.8) containing 0.6 mM EDTA. After a 30-min incubation at 25°C, 20 μl of 20 mM luminol and 11.6 U ml^−1^ horseradish peroxidase were injected, producing a light emission. The intensity of this emission was assumed to be proportional to the remaining quantity of H_2_O_2_. The CAT activity in the test samples was quantified by constructing standard curves using purified bovine liver CAT (Sigma-Aldrich) dissolved in the PBS-Triton buffer and expressed as U mg protein^−1^ (1 U corresponds to 1 μM H_2_O_2_ consumed min^−1^).

The GST activity was measured using the microplate spectrophotometric assay (Habig and Jakoby, 1981; Frasco and Guilhermino, 2002). The method is based on 1 mM glutathione (GSH) conjugation with 1 mM of 1-chloro-2.4 dinitrobenzene (CDNB) measured as absorbance decrement at 340 nm. The measurements were taken every 20 s during the first 5 min using 100 μl of the homogenate and 200 μl of the reaction solution [10 mM GSH in phosphate buffer (0.1 M, pH 6.5)] and 60 mM CDNB in ethanol using FLUOstar Optima with absorbance configuration. Enzyme activity values were expressed as U mg protein^−1^, where 1 U is the enzyme quantity necessary to conjugate 1 pmol CDNB min^−1^.

### Protein Concentration in the Homogenate

All enzymatic activities were normalized to the protein concentration in the respective sample. Protein concentrations were determined by a microplate fluorometric assay using the NanoOrange Protein Quantification Kit (Molecular Probes, Inc. Eugene, OR) with bovine serum albumin standards (Jones et al., 2003). In brief, 10 μl of the homogenate were diluted in NanoOrange working solution to achieve a final volume of 130 μl. Samples were incubated at 95°C for 10 min and cooled to room temperature for 25 min (light protected). Fluorescence was measured with FLUOstar Optima (filters: 485 nm for excitation and 590 nm for emission) reader and black solid flat-bottom microplates (Greiner Bio-One GmbH) with an integration time of 1 s.

### Quantitation of Individual RNA Content

We used individual RNA content in CVI copepods as a measure of condition and short term growth potential; this proxy has been successfully applied for both *A. bifilosa* and *E. affinis* growth assessment in the field (Höök et al., 2008) and experimental (Gorokhova and Engström-Öst, 2009) studies. The body RNA content in the copepod samples was quantified using microplate fluorometric high-range RiboGreen (Molecular Probes, Inc. Eugene, OR) assay after extraction with N-laurylsarcosine followed by RNase digestion (Gorokhova and Kyle, 2002). Fluorescence was measured in duplicates for each sample, standard, and negative control using FLUOstar Optima (filters: 485 nm for excitation and 520 nm for emission) reader and black solid flat-bottom microplates (Greiner Bio-One GmbH).

### qPCR-Based Gut Content Analysis

To quantify *Nodularia* and *Rhodomonas* in copepod guts (i.e., gut content, GC), a qPCR assay was applied. A 201 bp fragment of *Nodularia* 16S rDNA was amplified using NTS primer, TGTGATGCAAATCTCA(C/A)A (Moffitt et al., 2001) and universal 16S rRNA reverse primer 1494Rc, TACGGCTACCTTGTTACGAC (Neilan et al., 1997) following the established protocols (Gorokhova and Engström-Öst, 2009; Engström-Öst et al., 2011). A 213-bp fragment of *Rhodomonas* 18S rDNA was amplified using Rhod 1450F primer, GCGCGCTACACTGATGAATGC, and Rhod 1662R primer, TTTCACCGGACCATTCAATCG (Troedsson et al., 2009). For DNA extraction, 50 μl of 10% Chelex were added to a copepod sample and incubated for 3 h at 65°C. After spinning in a centrifuge (2 min at 12000 *g*), the supernatant (30 μl) was transferred to a clean tube and stored at 4°C for no more than 24 h. To prepare the standards, defined volumes of exponentially growing *Nodularia* and *Rhodomonas* cultures were filtered onto GF/F filters and dried at 50°C for 24 h (~1–2 mg DW per filter). Using a punch set, a 4 mm circle was sub-sampled from each filter, weighed, and DNA was extracted using the Chelex method. The DNA yield per sample was quantified using PicoGreen™ dsDNA quantitation kit (Molecular Probes, Eugene, OR) and contribution of DNA to DW was determined for each prey, 0.75 ± 0.05% for *N. spumigena* and 1.03 ± 0.08% for *R. salina* (mean ± SD; *n* = 5). These percentages were assumed to remain constant during the experiment. From each experimental unit, five individuals were analyzed in bulk and the amount of prey DW individual^−1^ was calculated using the respective percentages of DNA and error propagation rules.

Five-point standard curves were generated using 10-fold dilutions of the extracted DNA; duplicate negative controls (water) were included in all runs. Duplicate qPCR reactions were performed using StepOne real-time cycler (Applied Biosystems) and the QuantiTect SYBR Green PCR Kit (QIAGEN). For qPCR analysis of the copepod samples, the second-lowest concentration of standard DNA was added to all samples to ensure sample concentrations occurred within the standard curve. The measured concentration of samples was later adjusted to account for this added DNA. Amplifications were performed in a 25-μl reaction mixture with an initial denaturing step of 15 min at 95°C, and 40 cycles of 30 s at 94°C, 30 s at 53°C for *Nodularia* and 57°C for *Rhodomonas*, and 30 s at 72°C. An endpoint melt-curve analysis was generated after each run and analyzed to ensure non-specific PCR products’ absence; amplification efficiencies were 92–101%, with *R*^2^ > 0.98. To verify the authenticity of the PCR products, a random selection was purified using the Nucleo-Spin® Extract Kit (Macherey-Nagel), sequenced using ABI 3730 PRISM® DNA Analyzer at KIGene (Karolinska Institute, Stockholm, Sweden), and aligned with AY075067 and EU926158 (GenBank) for *N. spumigena* and *R. salina*, respectively.

### Statistics

In all statistical analyses, biochemical, molecular, and physiological variables measured in each experimental unit, post-exposure were analyzed using Statistica v. 8.0 for Windows (StatSoft Inc. 2001). For each variable and each copepod species, treatment effect (*diet*; two levels: mixed *Nodularia*/*Rhodomonas* vs. *Rhodomonas*), food availability (*food concentration*; five levels), and their interaction were evaluated using a two-way ANOVA followed by Bonferroni’s multiple comparison tests. When F-ratio statistics detected a significant interaction effect, a *t*-test was used for pair-wise comparison of treatment means. For linear statistics, log (x + 1) transformation was applied to RNA, EPR, VE, GC values, and enzyme activities to improve normality and homogeneity, while egg viability values (VE%) were arcsine square-root transformed.

Further, we used generalized linear models (GLZ) on untransformed values with gamma distribution and log link function to test for the *diet* effects on the relationships between the enzyme activities in each copepod species and physiological variables (copepod RNA content, EPR, VE%, and GC; continuous predictors). To establish the significant drivers of the enzymatic responses, the best-fit model was obtained using the Model Building Module in Statistica *via* screening the optimal number and combinations of the covariates. The best-fit model selection was based on considerations for parsimony, AIC, and residuals distribution. The variance inflation factor (VIF) set to <3 was used to select non-collinear variables (Zuur et al., 2010).

## Results

### Effects of Prey Species and Food Concentration on Antioxidative Enzymes

**Figure 1:**
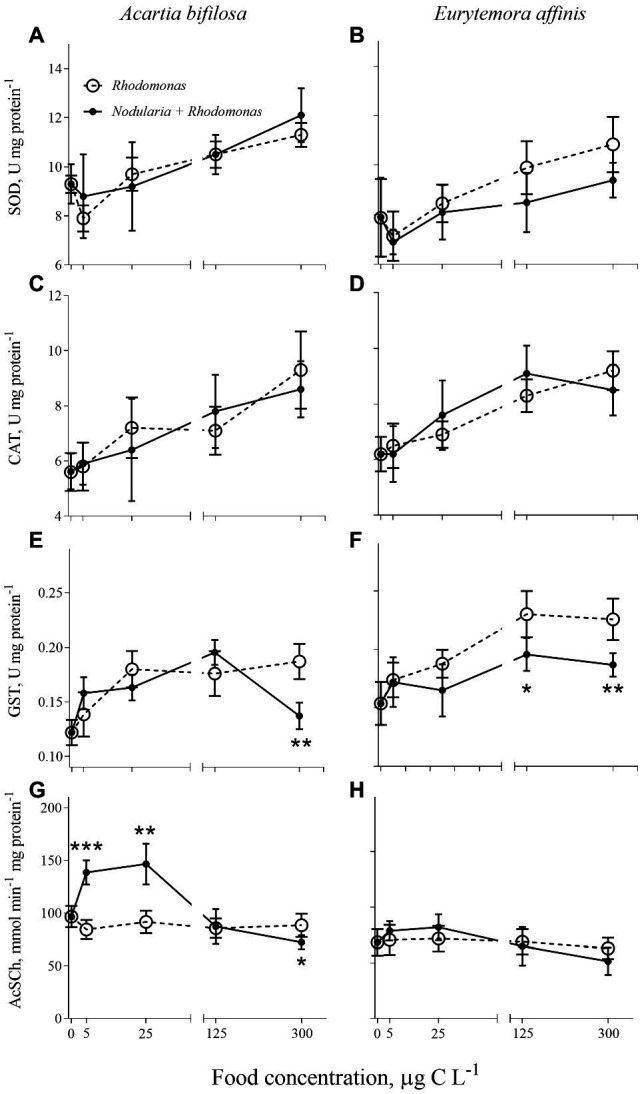
*Acartia bifilosa* **(A,C,E,G)** and *Eurytemora affinis* **(B,D,F,H)**: effects of diet (mixed *Rhodomonas salina* and *Nodularia spumigena* vs. *R. salina*) and food concentration (0–300 μg C L^−1^) on enzyme activities: **(A,B)** superoxide dismutases, SOD, U mg protein^−1^; **(C,D)** catalases, CAT, U mg protein^−1^; **(E,F)** glutathione S-transferases, GST, U mg protein^−1^; and **(G,H)** acetylcholinesterase (AChE) activity, nmol AcSC min^−1^ mg protein^−1^. The data are presented as mean ± SD (*n* = 5 in all cases); *p < 0.05, **p < 0.01, ***p < 0.001 when compared to the respective nontoxic control.

Regardless of the diet, SOD and CAT activities significantly increased with increasing food concentrations in both species (Figures 1A–D; Table 1). Also, GST activity significantly increased with food availability (Figures 1E,F; Table 1); however, in *A. bifilosa* fed *Nodularia*, this increase occurred only below150 μg C L^−1^, followed by a decrease at 300 μg C L^−1^ (Figure 1E). The decrease at the highest food concentration was significant compared to both non-toxic control (*t*-test: *t*_8_ = 4.954, *p* < 0.001) and the next highest concentration (*t*_8_ = 7.032, *p* < 0.0001). Moreover, significant positive correlations between all three antioxidant enzymes were observed in both copepods (Spearman rank correlation, 0.42–0.45 and 0.56–0.64 in *A. bifilosa* and *E. affinis*, respectively; *p* < 0.05 in all cases). The *diet* effect was significant only for GST in both copepods and marginally significant for SOD in *E. affinis*, with higher activities observed in the *Rhodomonas* monodiet (Table 1).

**Table 1:** Two-way ANOVA; sigma-restricted parameterization, effective hypothesis decomposition: *diet* (*Nodularia/Rhodomonas* vs. *Rhodomonas*) and *food concentration* (0, 5, 25, 125, and 300 μg C L^−1^) effects on SOD, CAT, GST (U mg protein^−1^), and AChE (nmol AcSCh min^−1^mg protein^−1^) activities in copepods.

Effect	*df*	*Acartia*		*Eurytemora*	
		*F*	*p*	*F*	*p*
**SOD**					
Food concentration	4	8.49	**<0.001**	9.04	**<0.001**
Diet	1	0.46	0.501	3.70	0.061
Diet × Food concentration	4	0.56	0.689	0.95	0.444
**CAT**					
Food concentration	4	11.71	**0.001**	17.91	**<0.001**
Diet	1	0.16	0.659	0.12	0.732
Diet × Food concentration	4	0.59	0.644	1.508	0.218
**GST**					
Food concentration	4	20.68	**<0.001**	16.16	**<0.001**
Diet	1	1.37	0.243	12.81	**0.001**
Diet × Food concentration	4	6.095	**0.001**	2.17	0.089
**AChE**					
Food concentration	4	14.65	**<0.001**	3.22	**0.022**
Diet	1	24.10	**<0.001**	0.30	0.582
Diet × Food concentration	4	15.38	**<0.001**	1.61	0.190

Significant effects (*p* < 0.05) are in boldface.

### Effects of the Diet and Food Concentration on AChE

A bell-shaped response in AChE activity to the food concentration in the mixed diet was observed in both copepods (Figures 1G,H). At low food availability (5–25 μg C L^−1^), the mixed diet stimulated AChE activity, with values increasing up to 53 and 15% in *Acartia bifilosa* and *Eurytemora affinis*, respectively. The increase was significant in *A. bifilosa* when compared to the starved control (*t*_8_ = 6.617, *p* < 0.001) and *Rhodomonas* monodiet (*t*_8_ = 5.997, *p* < 0.0003), but not in any treatment with *E. affinis* (*p* > 0.24 in all cases). At the highest mixed-diet concentration, AChE activity decreased relative to the respective non-toxic control (Figures 1G,H); the decrease was significant in *A. bifilosa* (*t*_8_ = 2.518, *p* < 0.04) but not in *E. affinis* (*p* > 0.17 in all cases).

### Effects of the Diet and Food Concentration on Feeding, Growth, and Reproduction

**Figure 2:**
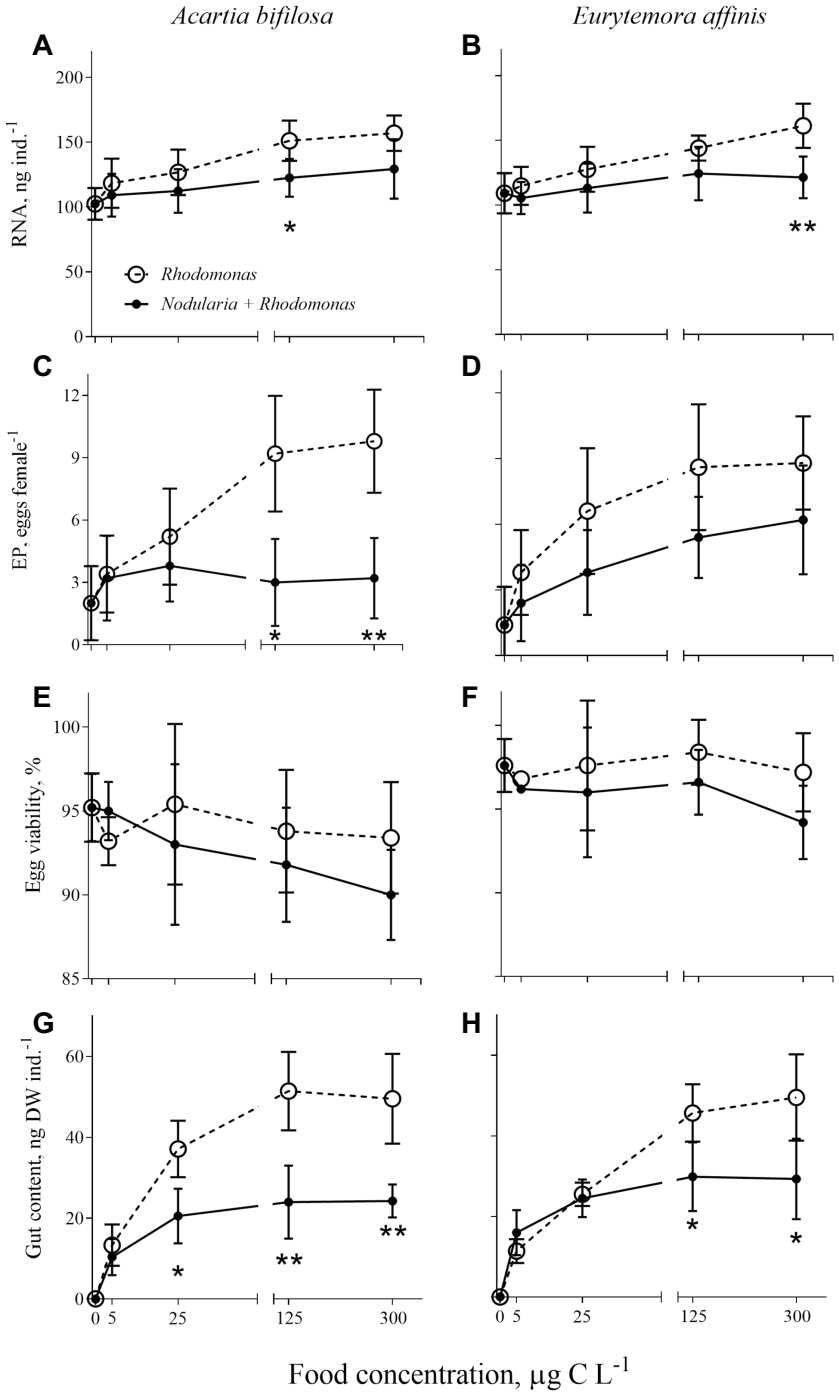
*Acartia bifilosa* **(A,C,E,G)** and *Eurytemora affinis* **(B,D,F,H)**: effects of diet (mixed *R. salina* and *N. spumigena* vs. *R. salina*) and food concentration (0–300 μg C L^−1^) on **(A,B)** individual RNA content, ng female^−1^; **(C,D)** egg production, EP, egg female^−1^; **(E,F)** egg viability, %; and **(G,H)** gut content, ng DW ind.^−1^. The data are presented as mean ± SD (*n* = 5 in all cases); *p < 0.05, **p < 0.01, ***p < 0.001 when compared to the respective nontoxic control.

As expected, individual RNA content, EPR, and GC values responded positively to increased food concentration (significant in all cases; Table 2; Figure 2). Moreover, the adverse effects of *Nodularia* on all these variables were significant in both species (*p* < 0.006 in all cases; Table 2). In *A. bifilosa*, the difference in the EPR between the copepods fed mixed and mono-*Rhodomonas* diets increased with increasing food concentrations (Figures 2C,D); this was reflected in the significant *diet × food concentration* interaction term (Table 2). Moreover, significant interactions for GC indicated that differences between the diets were not consistent for different food concentrations in both copepods (Table 2), increasing at higher food concentrations (Figures 2G,H).

**Table 2:** Two-way ANOVA; sigma-restricted parameterization, effective hypothesis decomposition: *diet* (*Nodularia/Rhodomonas* vs. *Rhodomonas*) and *food concentration* (0, 5, 25, 125, and 300 μg C L^−1^) effects on individual RNA content (RNA, ng ind^−1^), egg production (EP, egg female^−1^), viability (%EV), recruitment (viable egg female^−1^), and gut content (GC, ng DW ind^−1^) in copepods.

Variables	*df*	*Acartia*		*Eurytemora*	
		*F*	*p*	*F*	*p*
**RNA**					
Food concentration	4	9.24	**0.001**	6.33	**<0.001**
Diet	1	11.59	**0.002**	10.16	**0.002**
Diet × Food concentration	4	1.34	0.270	1.178	0.135
**EP**					
Food concentration	4	6.41	**0.001**	11.31	**<0.001**
Diet	1	18.69	**<0.001**	8.46	**0.006**
Diet × Food concentration	4	4.78	**0.003**	0.899	0.473
**Egg viability**					
Food concentration	4	1.04	0.398	1.143	0.3503
Diet	1	1.01	0.321	4.614	**0.0378**
Diet × Food concentration	4	0.69	0.658	0.6318	0.6434
**Recruitment**					
Food concentration	4	5.05	**0.0022**	**12.91**	**<0.001**
Diet	1	10.01	**0.0029**	**5.28**	**0.0268**
Diet × Food concentration	4	2.26	0.0797	0.35	0.8416
**GC**					
Food concentration	4	49.33	**<0.001**	67.63	**<0.001**
Diet	1	93.03	**<0.001**	12.85	**0.001**
Diet × Food concentration	4	8.64	**0.001**	7.217	**0.001**

Significant effects (*p* < 0.05) are in boldface.

### Effects of Physiological Variables on Enzyme Activities

In *Acartia bifilosa*, significant positive effects of *Nodularia* on the antioxidant enzyme (SOD, CAT, and GST) and AChE activities were observed when accounted for the confounding effects of growth status (SOD and CAT) and/or feeding (CAT and GST; Table 3). In *Eurytemora affinis*, this was true for CAT activity only (Table 3). GC was a significant predictor of SOD, CAT, and GST values in the best-fit models for both species, indicating a positive effect of food intake on enzyme activities.

**Table 3:** Best-fit GLZ models for relationships between enzyme activities (AChE, SOD; CAT and GST) as response variables and diet (*Nodularia/Rhodomonas* vs. *Rhodomonas*) as the categorical independent factor and individual RNA content (RNA), egg production (EP), egg viability (EV%), and gut content (GC) as possible continuous predictors in *Acartia bifilosa* and *Eurytemora affinis*.

Variables	*Acartia*			*Eurytemora*		
	Estimate	Wald statistics	*p*	Estimate	Wald statistics	*p*
**SOD**						
Diet	0.0017	4.03	**0.044**			
RNA	0.0029	7.90	**0.005**	0.0035	7.37	**0.006**
GC	0.066	2.48	0.112	0.0036	7.23	**0.007**
**CAT**						
Diet	0.0730	6.21	**0.013**	0.0719	9.76	**0.002**
RNA				0.0038	11.05	**0.001**
EV%	−1.544	5.88	**0.016**			
GC	0.0061	23.30	**<0.001**	0.0031	5.54	**0.019**
**GST**						
Diet	0.057	7.34	**0.007**			
GC	0.0052	13.45	**0.004**	0.0537	23.96	**<0.001**
**AChE**						
Diet	0.0961	5.96	**0.011**			

Significant effects (*p* < 0.05) are in boldface; only predictors contributing to the winning models are shown.

### Egg Viability

Egg viability was significantly lower in *A. bifilosa* compared to *E. affinis*, decreasing with increased food concentration (GLZ; *diet*: *t*_48_ = 2.95, *p* < 0.004; *copepod species*: *t*_48_ = 3.38, *p* < 0.001), whereas *diet* × *copepod species* interaction was never significant (Table 2) indicating that these trends were consistent between the copepod species. Together, lower egg production and viability translated into significantly lower recruitment estimated as a product of EPR and EV% in copepods fed mixed diet than those fed with *Rhodomonas* monodiet (Table 2).

Of all enzyme activities measured, the only significant predictors for the proportion of viable eggs were CAT (*Acartia*: Wald statistics = 5.39, *p* < 0.02) and GST (*Eurytemora*: Wald statistics = 4.42, *p* < 0.03), with negative and positive effects, respectively. The recruitment was significantly predicted by a combination of RNA as a positive predictor (Wald statistics = 45.21, *p* < 0.0001) and SOD as a marginally significant negative predictor (Wald statistics = 2.84, *p* > 0.08), with no significant effects of either species or diets.

## Discussion

Cladocerans and copepods are test organisms in ecotoxicological testing, including biomarker applications. The challenge is to understand the biomarker variability in these highly adaptable grazers under variable environmental conditions, including food concentrations and foods with high levels of natural toxins and biologically active metabolites. Most ecotoxicological surveys employing biomarkers to identify the biological effects of contaminants ignore the natural toxins in the environment as well as food availability and growth status of the test animals. We addressed this issue by assessing biomarker (antioxidant and AChE activities), feeding, and growth responses in the Baltic copepods to feeding conditions representative of heavy cyanobacteria bloom conditions in the northern Baltic proper. The primary focus was on evaluating food availability, intake, and growth as confounding variables for the biomarker responses and additional effects of species and diet.

We induced antioxidant and growth responses in the copepods *Acartia bifilosa* and *Eurytemora affinis* by manipulating food availability and diet composition, i.e., mixed diet dominated by toxic *Nodularia spumigena* or the high-quality prey *Rhodomonas salina*. We observed that SOD, CAT, and GST activities increased with increasing food availability regardless of the diet, with no significant diet effect when nominal food concentration was used as a covariate in the regression analysis (Figure 1; Table 1). However, the pro-oxidative effects of *N. spumigena* became highly significant when antioxidant enzyme activities were compared between the diets while also accounting for the variation in the metabolic responses, i.e., feeding, growth status, and egg production of the animals (Table 3). These findings emphasize the importance of complementary assessment of metabolic status in the test species when applying oxidative stress biomarkers in ecological and ecotoxicological studies. They also indicate that grazers feeding on toxic cyanobacteria (and perhaps other phytoplankton species producing bioactive secondary metabolites) have elevated ROS production. Thus, the imbalance between the pro-oxidant processes and the enzymatic antioxidant activities may increase with the increasing contribution of such foods to the diets.

Of the two copepods, *E. affinis* was less affected by the diet than *A. bifilosa*; this holds for both enzymatic and metabolic responses (Figures 1 and 2). Moreover, at food concentrations ≤125 μg C L^−1^, which are below average bloom values in the Baltic Sea (Jaanus et al., 2011), *E. affinis* had a similar feeding rate at different diet treatments as suggested by the gut content response, whereas *A. bifilosa* was feeding less, avoiding the cyanobacteria (Figure 2H). These findings are in line with the reported differences in the adaptation strategies to the cyanobacteria blooms between the two species in the Northern Baltic Proper. In *E. affinis* co-occurring with cyanobacterial blooms, positive effects of *Nodularia* ingestion on growth, reproductive output, and naupliar survival have been reported (Hogfors et al., 2014; Motwani et al., 2017), indicating that all ontogenetic stages of this species are well-adapted to the cyanotoxins and can efficiently utilize toxin-producing cyanobacteria. Recently, the mechanism for the species-specific growth responses to hepatotoxin-producing cyanobacteria was proposed (Gorokhova et al., 2021). This mechanism is based on environmental and genetic adaptation in the copepod microbiome that harbors higher abundances of toxin-degrading bacteria in *E. affinis* than in *A. bifilosa*. The adaptation has evolved in response to the regular co-occurrence of these copepods with blooms of *N. spumigena* and, perhaps, microcystin-producing Baltic cyanobacteria, such as *Dolichospermum* (Johansson et al., 2004). The differential responses for biomarkers and physiological variables observed here are in line with the enhanced capacity of *E. affinis* to handle the cyanobacteria-rich diet and maintain growth. Thus, low-to-medium bloom conditions are likely to stimulate population growth in this copepod, and it is likely that *E. affinis* relies on these blooms to maintain its production in the Baltic Sea.

Despite the growing applications of oxidative stress diagnostics in ecology, we are only beginning to understand the complexity of extrinsic and intrinsic factors behind oxidative stress in different organisms. We expected to find a positive association between the antioxidant response and feeding, because increased caloric intake facilitates the pro-oxidative processes (Sohal and Weindruch, 1996), i.e., the biochemical reactions directly related to feeding and metabolic rates. The superoxide anion (O_2_¯) derived from oxidative respiration is the most common ROS and the primary species directly generated in the mitochondrial respiratory chain by converting molecular oxygen to O_2_¯ by single-electron transfer (Fleury et al., 2002). Metalloenzymes SOD catalyse the dismutation of O_2_¯ to H_2_O and H_2_O_2_, which is then detoxified by CAT and glutathione peroxidase (Halliwell, 2006). Thus, the SOD–CAT system is often referred to as the first defense line against oxygen toxicity; moreover, the concerted response of these enzymes is sometimes used to indicate coping with elevated ROS production in ecotoxicological studies. The coordinated response manifested as correlative changes in SOD and CAT activities was reported in invertebrates exposed to chemical contaminants (Gorokhova et al., 2013). In line with that, we found that SOD and CAT activities were positively correlated in both copepod species, suggesting a common mechanism for their induction and regulation. Moreover, both enzymes increased across food availability gradients (Table 1; Figure 1) in concert with increasing food intake, individual RNA levels, and egg production (Table 3). This increase provides yet another evidence that elevated metabolic activity due to variability in feeding is associated with higher production of ROS and consequently induction of antioxidant defenses. In crayfish, ingestion of dietary proteins in excess of metabolic amino acid requirements increases superoxide anion production, resulting in elevated lipid peroxidation in gills (Zenteno et al., 2008). Moreover, not only food quantity but also its quality may affect antioxidant responses as suggested by a decreased activity of CAT and GST in the cladoceran *Daphnia commutate* experiencing dietary phosphorus limitation (Balseiro et al., 2008) and by a positive correlation between GST and body condition assessed by C:P ratio in copepods (Souza et al., 2010). In line with the latter study, GST activity in *E. affinis* was driven by food consumption only (Table 3). Therefore, when applying the biomarker approach in laboratory and field investigations to assess animal health status, measuring growth and metabolic status in the test specimens or using appropriate biomarkers for these physiological variables is also crucial.

Although high food availability had a more substantial pro-oxidative effect than the toxic cyanobacterium in our study, oxidative stress was also a possible biochemical mechanism of the cyanobacterial toxicity, especially in *Acartia* (Table 2). Earlier observed *in vivo* oxidative stress effects induced by purified microcystins or crude cyanobacterial extracts include depletion of intracellular glutathione, changes in mitochondrial function and apoptosis induction, lipid peroxidation, and modulations of GST, CAT, SOD, and glutathione peroxidase (Pietsch et al., 2001; Ding and Ong, 2003; Zhang et al., 2008). When we accounted for the variability in food consumption, the diet effects on the antioxidant enzyme activities were significant for SOD, CAT, and GST in *Acartia*, whereas only CAT was significant in *Eurytemora* (Table 3). Therefore, our observations are in agreement with the known pro-oxidative mode of action of various non-ribosomal peptides produced by cyanobacteria.

The GSTs are a ubiquitous multigene enzyme superfamily involved in detoxifying xenobiotics and natural toxins, such as microcystins (Wiegand and Pflugmacher, 2005), by catalyzing their conjugation with glutathione and making the parent compound more water-soluble. Additionally, GSTs are involved in removing reactive organic hydroperoxides, such as lipid peroxidation products (Pflugmacher et al., 1998; Beattie et al., 2003), thus providing defenses against ROS and their toxic metabolites. However, whereas exposure to the pure microcystins induced GST in *Danio rerio*, the enzyme activity was suppressed after exposure to the crude extract of *Microcystis aeruginosa* dominated bloom, probably due to the presence of lipopolysaccharides that may reinforce the effects of microcystins by inhibiting the activity of GSTs (Pietsch et al., 2001; Best et al., 2002). In line with this, a bell-shaped response in GST activity was observed in *A. bifilosa*. This species was significantly higher in the mixed-diet treatment compared to the *Rhodomonas* monodiet, with a significant increase at the cyanobacterial concentration of up to 125 μg C L^−1^ followed by ~30% drop at the highest concentration (Figure 1E). The reduction in GST availability due to the lipopolysaccharide production reported for zebrafish embryos (Best et al., 2002) may deleteriously affect the ability of organisms to detoxify microcystins, presumably through decreased utilization of glutathione for conjugation reactions, although other interpretations of the observed GST dynamic are possible. While the glutathione system is an essential intracellular redox buffer, preventing oxidative injury, it is also a key enzyme in various cellular functions, including protein and DNA synthesis, amino acid transport, metabolism, and cell growth. Moreover, in addition to GST, many other metabolic enzymes are glutathione-dependent, which may, at least partly, explain the sustained dynamics of both enzymatic and organismal responses at higher food concentrations (Figures 1 and 2). Accordingly, low retention of ingested cyanobacteria toxins by copepods is usually observed (Guisande et al., 2002; Kozlowsky-Suzuki et al., 2003) and could be explained by detoxification.

Contrary to the expected unimodal inhibition of AChE with increasing cyanobacteria concentrations, an unusual response of AChE increasing up to >50% at low (≤25 μg C L^−1^) levels of the cyanobacterium was observed in *A. bifilosa*, whereas no significant response was detected at 125 and 600 μg C L^−1^ (Figure 1B). The AChE activity is a popular biomarker to detect a response to organophosphate, carbamate, and metal exposure, with the enzyme inhibition being a sign of chemically-induced neurotoxicity (Sarkar et al., 2006). The commonly observed inhibition of AChE is linked directly with the mechanism of toxic action, *viz*. irreversible or reversible binding to the catalytic site of the enzyme and potentiation of cholinergic effects (Sarkar et al., 2006). However, chemically-induced increase in AChE levels has also been observed in various species, including microcrustaceans and other test organisms commonly used in ecotoxicology (Srivatsan, 1999; Jemec et al., 2007; Gorokhova et al., 2013; Eriksson Wiklund et al., 2014), which may be related to various roles of AChE responding to many external stimuli other than in cholinergic neurotransmission (Soreq and Seidman, 2001). Moreover, in *M. affinis* exposed to contaminated sediment under hypoxic conditions, AChE correlated positively with mortality, indicating that elevated AChE activities are associated with physiological stress (Gorokhova et al., 2010, 2013). Therefore, the cyanobacteria-induced AChE stimulation in copepods and possibly other crustacean grazers should be considered when interpreting field measurements of this biomarker in ecotoxicological surveys.

Our study was conducted using copepods collected in the Northern Baltic Proper, where toxic cyanobacterial blooms occurred over 7,000 years (Bianchi et al., 2000). Surface aggregations of summer cyanobacteria, including *N. spumigena* (Hajdu et al., 2007), have high cell density and toxin concentrations, thus increasing the potential toxicity to zooplankton. Therefore, the copepods in this ecosystem are evolutionarily adapted to cyanotoxins and bioactive compounds produced by this cyanobacterium. However, due to more recent anthropogenic activities, this area is also greatly influenced by direct and indirect toxic contaminants, harmful to biota and humans. Of great interest for ecological assessment for contaminant effects in biota is to provide evidence for how anthropogenic pressures impair essential physiological functions of the Baltic fauna. However, our study shows that toxin-producing algae may act as confounding stressors and cause biological responses (even irreversible damage), including the biomarkers commonly used for biological effect assessment in the context of chemical exposure. Therefore, comprehensive experimental studies with combined exposure to cyanotoxins and chemical mixtures relevant for specific habitats are needed to select informative test species, biomarkers, and covariates, such as food intake and growth-related variables, to use biomarker approaches in the environmental assessment of chemical stressors.

## Data Availability Statement

The raw data supporting the conclusions of this article will be made available by the authors, without undue reservation.

## Author Contributions

EG designed the experiments. EG and RE analyzed the results and wrote the manuscript. All authors contributed to the article and approved the submitted version.

## Conflict of Interest

The authors declare that the research was conducted in the absence of any commercial or financial relationships that could be construed as a potential conflict of interest.

## Publisher’s Note

All claims expressed in this article are solely those of the authors and do not necessarily represent those of their affiliated organizations, or those of the publisher, the editors and the reviewers. Any product that may be evaluated in this article, or claim that may be made by its manufacturer, is not guaranteed or endorsed by the publisher.
